# Parental Perceptions of Benefits and Risks Concerning Mental Health Biomarker Testing for Adolescents

**DOI:** 10.1007/s10597-026-01601-0

**Published:** 2026-02-25

**Authors:** Kaustubh Kishor Jadhav, Kirsi Kuuru, Eeva Aromaa, Aino-Kaisa Piironen, Päivi Eriksson, Katja M. Kanninen, Tommi Tolmunen

**Affiliations:** 1 A. I. Virtanen Institute for Molecular Sciences, Faculty of Health Sciences, University of Eastern Finland, Kuopio, Finland; 2 Business School, Faculty of Social Sciences and Business Studies, University of Eastern Finland, Kuopio, Finland; 3 Institute of Clinical Medicine, Department of Medicine, Faculty of Health Sciences, University of Eastern Finland, Kuopio, Finland; 4 Department of Adolescent Psychiatry, Kuopio University Hospital, Kuopio, Finland

**Keywords:** Biomarker, Testing, Attitude, Adolescent, Mental health, Perception

## Abstract

Psychiatry currently lacks clinically relevant biomarkers, though recent developments in this field have shown promise. Nevertheless, a noticeable gap exists in understanding parental perspectives regarding such technologies. Thus, this study aimed to investigate parental perceptions about mental health biomarker (MHB) testing for adolescents, as they play a pivotal role in guiding decisions about their children’s healthcare. An online survey was conducted in Finland, targeting 1500 parents of adolescents aged 10–17. A 20-item questionnaire was used to assess parental perceptions of the benefits and risks associated with MHB testing. Psychometric measures were applied to validate the questionnaire, and a Multivariate Analysis of Variance (MANOVA) was performed to examine parental perspectives across various demographic factors. Based on 174 questionnaire responses, parents were modestly supportive of the perceived benefits and expressed marginally lower concern about the possible risks associated with MHB testing. MANOVA indicated that some demographic variables were significantly associated with perceived benefits and risks. Notably, higher perceived benefits of adolescent MHB testing were reported by relatively younger parents and those with an undergraduate degree. Additionally, parents with a family history of mental health problems reported greater perceived benefits and lower perceived risks. The findings suggest that perceptions of the benefits and risks of MHB testing differ, with demographics influencing these perceptions. However, further research is warranted to explore the impact of these perceptions on the utility and acceptance of MHB testing among parents and adolescents.

**Supplementary Information:**

The online version contains supplementary material available at 10.1007/s10597-026-01601-0.

## Introduction

The incidence of mental health problems (MHP) has increased globally, particularly among school-aged children, and especially in the aftermath of the COVID-19 pandemic​ (Bommersbach et al. 2023). This trend has raised serious concerns, as the prevalence of MHPs has surged over the past decade, drawing attention to the importance of early diagnosis and the development of effective testing methods. This is where biomarkers come into focus, particularly those with the potential to predict future risks of MHPs (de Sousa Maciel et al. 2023; Mongan et al. 2021). Biomarkers are defined as characteristics that are objectively measured and evaluated as indicators of normal biological processes, pathogenic processes, or pharmacologic responses to a therapeutic intervention​ (Atkinson et al. 2001)​. Biomarkers indicating the potential for developing a disease or a medical condition in an individual who does not currently have a clinically apparent disease or a medical condition​ (Califf 2018) may serve as valuable tools for early diagnosis​. Research into biomarkers has gained significant attention in recent years, intending to develop mental health biomarker (MHB) testing to identify individuals at risk of developing MHPs (Chan et al. 2015; de Sousa Maciel et al. 2023).

Adolescence is a critical phase of brain development and is often identified as the period when the first incidence of MHPs usually occurs (Fuhrmann et al., 2015). Research evidence indicates that many MHPs experienced in adulthood have begun in early or late adolescence, underscoring the significance of this developmental stage. The onset of MHPs frequently occurs before the age of 18 on a global scale (Scheiner et al., 2022; Solmi et al., 2022). This highlights adolescence as a crucial period for early detection and implementation of interventions. Given the importance of this phase, biomarkers that predict the risk of developing MHPs could play a transformative role. With advances in biomarker technology, early identification of at-risk individuals could become feasible in the future. Adolescents, under 18 years, often require parental involvement in decision-making, as legal consent for testing varies across countries. Thus, the responsibility for making informed decisions largely falls on their parents/caregivers. This critical role places parents at the center of discussions about MHB testing.

Given the potential of biomarkers in predicting health outcomes, genetic testing has been developed to use biological markers to estimate an individual’s susceptibility to specific conditions. As such, genetic testing provides a relevant reference point for understanding parental perceptions of MHB testing. While genetic testing has been widely regarded as beneficial (Lim et al., 2017; Yamamoto et al., 2021), research lacks exploring similar perspectives for MHB testing. This underscores the importance of exploring whether similar perspectives prevail regarding MHB testing, particularly given their shared goal of early risk prediction and the influence of parental perceptions of benefits and risks on the utility of genetic testing (Tercyak et al., 2011). To date, research on MHBs has focused primarily on their discovery, validation, and clinical feasibility, with limited attention to the societal contexts in which these technologies will be implemented. Yet perceptions, acceptability, and real-world utility are critical determinants of successful translation from laboratory to clinical practice. In adolescent mental health, parental perspectives are especially influential, as parents serve as key decision-makers in settings where adolescents cannot provide informed consent. How parents interpret MHB testing, including the perceived benefits, risks, and uncertainties, may shape its adoption and clinical uptake. These views may be further influenced by media portrayals of biomarker research, which can amplify mistrust or unrealistic expectations. Examining parental perspectives, therefore, advances biomarker research by integrating social acceptability into early-stage innovation, supporting more responsible, feasible, and sustainable implementation of adolescent MHB testing. This study is a crucial early step in the pre-implementation phase of biomarker development, providing the necessary links to ensure this emerging technology is not only scientifically valid but also socially viable. Previous research on genetic testing has shown that parents are generally supportive of testing following its potential to aid in early detection and manage future medical complications​ (Levine et al., 2010)​. Caregivers reported that genetic testing provides clear diagnoses, improves treatment decisions, and supports better future healthcare planning (Zhou et al., 2023). These insights reflect a broader trend of optimism surrounding genetic testing’s role in advancing health and well-being. Given this context, exploring whether similar perceptions extend to MHB testing is essential.

So, building on existing beliefs and perceptions about genetic testing, our study aimed to explore how parents perceive the benefits and risks associated with MHB testing for adolescents. Furthermore, we examined whether demographic factors tend to influence these perceptions. This study offers initial insights into parental perceptions of the benefits and risks associated with MHB testing for adolescents. Our research contributes to understanding this underexplored area, which could be helpful for informing future policy and practical applications in the field of MHB testing.

## Methods

### Participants

Postal addresses for 1,500 parents of adolescents were obtained from the Digital and Population Data Services Agency to distribute survey materials. Eligibility criteria for this included: (1) having at least one adolescent aged 10–17 years, (2) being a native Finnish speaker, and (3) residing in the Kuopio region of Finland. Participants were selected using a random sampling procedure. The research team had no prior contact with the selected individuals. As Finnish is the primary language spoken in the Kuopio region, the questionnaire was administered in Finnish to ensure comprehension; accordingly, native Finnish speakers were included. Participation was voluntary, no incentives or prizes were offered, and written informed consent was obtained from all participants prior to participation.

### Questionnaire

​​At the end of 2022, a comprehensive literature search was conducted to identify research articles using the keywords “attitude”, “acceptance”, and “mental health (and biomarker) testing”. No suitable questionnaire was identified to fully address the research aims. Given the conceptual similarities between genetic testing and biomarker testing, both involving interventions designed to mitigate future risks, a second literature search focused on “genetic testing.“​ This led us to the article by Yusuf et al., which examined the perceived utility of biological testing for autism spectrum disorder (ASD) (Yusuf et al., 2020). Detailed information about our questionnaire design is provided in the supplementary file. Sociodemographic factors such as participant age, educational level, family annual income, number of adolescents (and their ages), family history of mental health problems (if applicable), and participants’ knowledge about MHPs were included in the final questionnaire (see supplementary file #2 & #5).

The online questionnaire was developed using the Webropol service at the University of Eastern Finland and was pseudonymized to ensure that no personal data, such as names, emails, or social security numbers were collected.

### Data Analysis

All analyses were conducted using IBM SPSS Statistics software (v29.0.2). Given that the questionnaire addressed a different research question and population than originally intended, we conducted an Exploratory Factor Analysis (EFA). The EFA utilized the Principal Axis Factoring method (Williams et al., 2010) with Promax rotation, consistent with the approach detailed in the original questionnaire, where all questions loaded onto a single factor, indicating correlation. Factor loading values < 0.5 were restricted to account for our smaller sample size and to enhance accuracy. Additionally, the Kaiser-Meyer-Olkin (KMO) Measure of Sampling Adequacy and Bartlett’s Test of Sphericity were calculated. To determine the number of factors to retain, parallel analysis and the scree plot test were used (O’connor, 2000; Osborne, 2014). After determining the optimal factor solution from EFA, the benefit and risk factor scores were saved using the regression method and retained for subsequent analyses. MANOVA was conducted to simultaneously compare perceptions of benefits and risks, assuming that these dimensions can be expressed concurrently, rather than as opposing endpoints on a spectrum. Bonferroni corrections were applied to adjust for the multiple comparisons being made. Demographic variables were included both individually and collectively to assess their independent and combined effects. Additionally, Pearson’s correlation, t-tests, and linear regression were utilized as needed to generate supplementary outputs. Responses in which participants selected “do not want to answer” for demographic items were treated as missing values (see supplementary material #4). Because the proportion of missing data was less than 5%, listwise deletion was applied across all analyses. Little’s Missing Completely At Random (MCAR) test was non-significant, indicating that the MCAR assumption was met.

The Ethical Statement for the study was provided by the University of Eastern Finland Committee on Research Ethics (No. 11/2023) in 2023.

## Results

### Demographics

Of the 1,500 invitations sent, 174 parents completed the survey, resulting in a response rate of approximately 12%. The mean age of participants was 45.3 ± 5.5 years, ranging from 31 to 60. The sample was predominantly female, with 76% identifying as female and 24% as male. In terms of educational attainment, 38% of participants held an undergraduate/bachelor’s degree, and 44% had a postgraduate degree (master’s/doctorate/equivalent). Regarding socioeconomic status, most participants (39%) reported family income exceeding €90,000, including social benefits.

Family composition was characterized by an average of two or fewer adolescents (aged 10–17 years) per household. Over 70% of participants reported that neither they themselves nor their adolescents had received any mental health diagnoses. However, 44% of participants acknowledged a family history of mental health problems. Detailed demographic information is presented in Table 1.

**Table 1:** Demographic characteristics of the participants represented as the number of participants and percentages

Demographics	Number of participants (*n*)	Percentages (%)
1. Education		
Pre-university	31	17.8
Undergraduate degree	66	37.9
Postgraduate degree	77	44.3
Missing	-	-
2. MHP (diagnosed)		
Participants*	44	25.3
Their adolescents*	37	21.3
Family members*	77	44.3
Missing**	02	0.4

*separate questions (each answered by 174 parents) **combined missing values

### Psychometric Analyses

The initial Cronbach’s alpha for all the questions in the benefits section was 0.90, and in the risks section was 0.84. After performing the EFA initially by restriction to eigenvalues, KMO Adequacy was 0.84, which indicates that sampling was adequate, and Bartlett’s Test of Sphericity was significant (χ2(190) = 1729.5, *p* < 0.001). Comparing data values with parallel analysis, a two-factor solution was most suitable for our 20 questions. This supported our choice of grouping questions into benefits and risks. When restricting factor loadings to 0.5, all risk questions loaded exclusively onto the second factor. While most benefit questions loaded onto the first factor, some demonstrated loadings greater than 0.7 on a different factor. Additionally, certain questions had loadings below 0.5. These items were evaluated for content validity in the context of MHB testing for adolescents. Questions with loadings below 0.5, those lacking content validity, and those with cross-loadings were excluded. Comprehensive details regarding this process are provided in the supplementary file #6. Ultimately, the benefits section comprised nine questions, and the risks section included six questions (see Table 2).

**Table 2:** Questionnaire after EFA and retaining a 2-factor solution

No.	Modified questions
B2	I would like my child to have biomarker tests regardless of the results
B3	I would like to know the results of biomarker tests related to my child’s mental health in the future
B8	Biomarker test results would help me prepare for my child’s future
B9	Biomarker tests would help me stay informed about future treatment options
B10	Biomarker test results would help me take better care of my child and family
B11	Biomarker test results could help prevent my child from developing other health problems
B12	The results of biomarker tests and their potential to prevent mental health problems would put my mind at ease about my other children’s risk of illness
B13	Biomarker test results would make me more watchful for my other children’s symptoms
B14	Biomarker tests would give me information about other mental health problems my child may develop
R1	Biomarker test results can undermine my freedom to decide about my child’s health
R2	Biomarker test results could stigmatize my family
R3	Biomarker test results can cause problems between me and my (ex) partner
R4	After biomarker tests, I may feel guilty that I may have passed on a biological risk to my child
R5	Biomarker test results may leave me living in uncertainty
R6	Biomarker test results may make me focus on mental health problems instead of what life has to offer

After refining to a two-factor solution, all retained questions had loadings exceeding 0.5. The benefits and risks factor scores were saved using the regression method. After EFA, Cronbach’s alpha for the benefits section was 0.87, and for the risks section was 0.84, indicating strong internal consistency.

### Perception of Benefits and Risks

The distribution of factor scores indicated that a slightly greater proportion of parents scored above the sample average on perceived benefits (*n* = 91) and below the sample average on perceived risks (*n* = 89), suggesting a marginally more favorable orientation toward benefits than risks. Additionally, composite mean scores derived from the Likert-scale items showed modestly higher perceived benefits (M = 3.60) and somewhat lower perceived risks (M = 2.60). A paired-samples t-test revealed a statistically significant difference between the two (t(173) = 9.91, *p* < 0.001). Overall, parents demonstrated a modest preference toward perceived benefits while expressing relatively limited concern about perceived risks.

#### The Effect of Education on Perceived Benefits and Risks

**Fig. 1:**
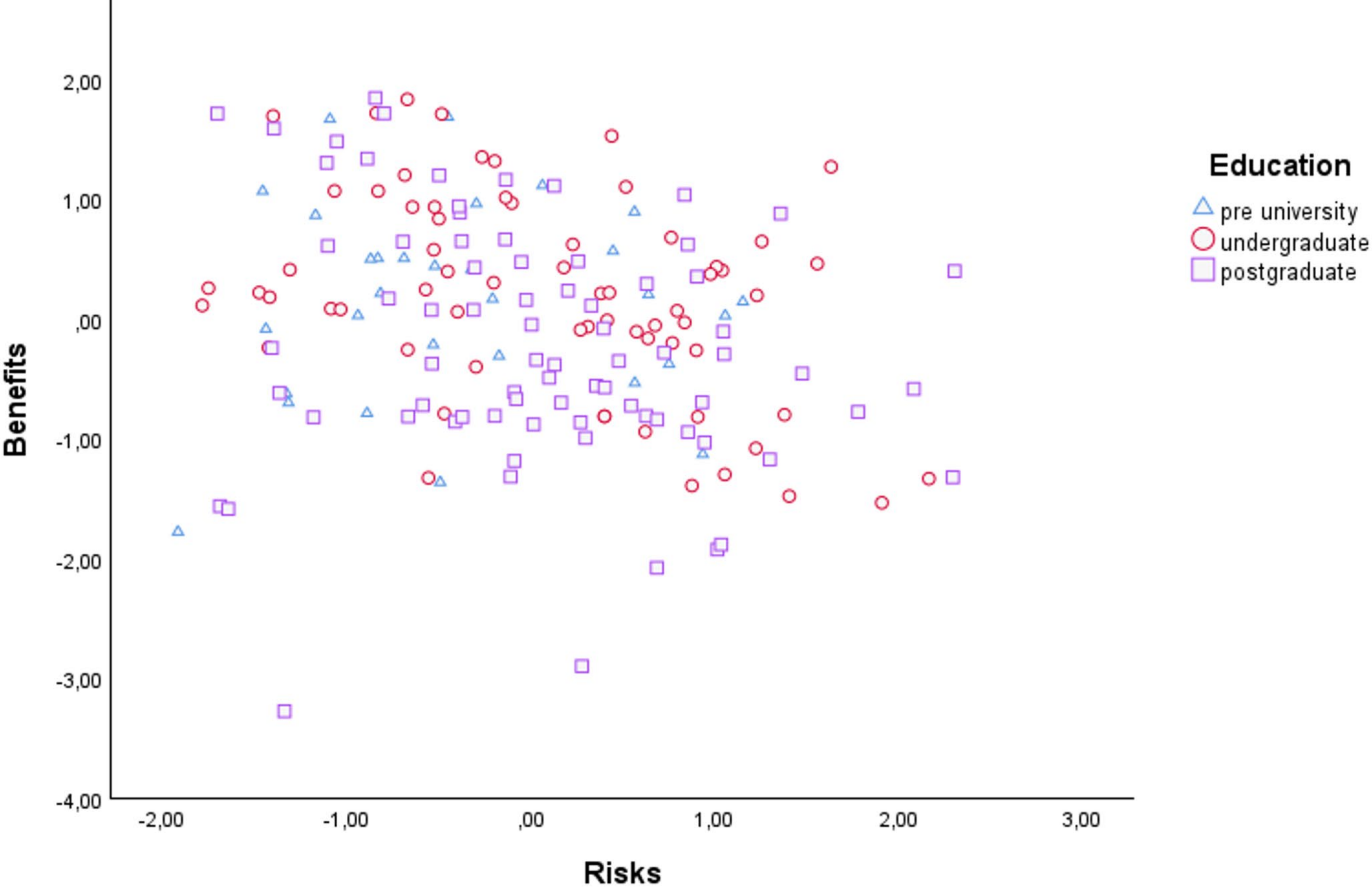
Scatter plot of benefits versus risks categorized by education levels

When MANOVA was performed with individual demographic factors, education was the only variable with a significant multivariate effect on parental perceptions (Pillai’s Trace = 0.077, F(4, 342) = 3.42, *p* = 0.009), and the assumption of homogeneity of variance-covariance matrices was met (Box’s M = 8.35, *p* = 0.22). Follow-up univariate analyses showed that education significantly influenced perceived benefits (F(2, 171) = 3.72, *p* = 0.026, partial η² = 0.04), but not perceived risks (*p* = 0.055). Although statistically significant, the observed effects were modest. Bonferroni-adjusted group comparison indicated that parents with an undergraduate degree reported significantly higher perceived benefits than those with a postgraduate degree (mean difference = 0.41, SE = 0.16, *p* = 0.028, 95% CI [0.03, 0.80]). No other comparisons were statistically significant. Additionally, linear regression, using postgraduate education as a reference, revealed that parents with an undergraduate degree reported significantly higher perceived benefits (β = 0.21, B = 0.41, SE = 0.16, t = 2.63, *p* = 0.009, 95% CI [0.10, 0.72]). Specifically, the benefits score was higher by 0.41 units for parents with an undergraduate degree, compared to those with a postgraduate degree. Scatter plots were created with benefits on the y-axis and risks on the x-axis to visualize the distribution based on educational qualifications, as shown in Fig. 1.

**Fig. 2:**
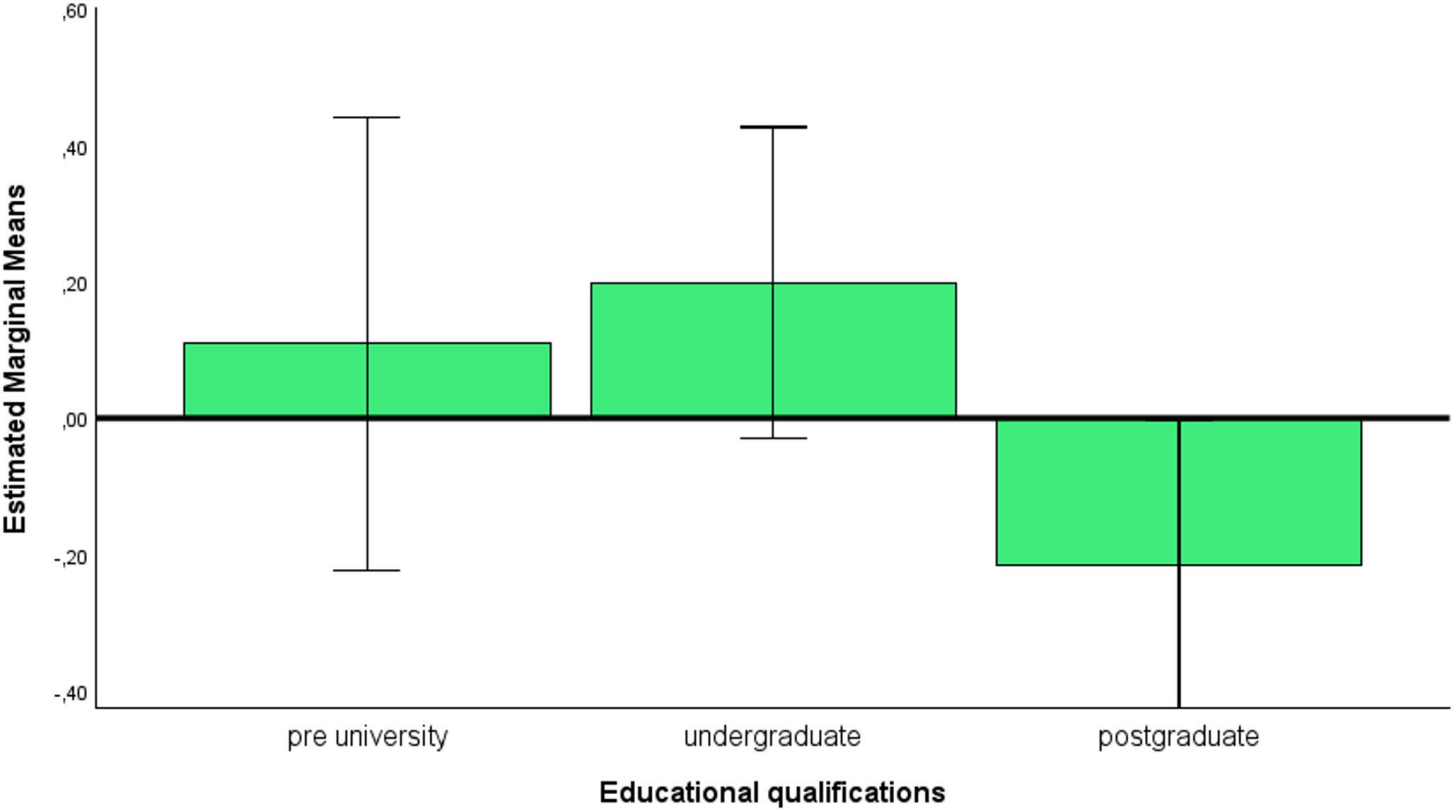
Bar graph of estimated marginal means of benefits by educational qualifications

Furthermore, a bar graph was created to visualize the perceived benefits (estimated marginal means) in relation to educational qualifications, as shown in Fig. 2.

#### The Effect of Age on Perceived Benefits and Risks

Since age was normally distributed, participants were divided into two age groups, considering the midpoint of the age range of the respondents: younger participants (30–45 years) and older participants (46–60 years). When MANOVA was conducted with all demographic variables entered collectively, age was the only factor showing a significant multivariate effect on parental perceptions (Pillai’s Trace = 0.048, F(2, 150) = 3.75, *p* = 0.026), and the assumption of homogeneity of variance-covariance matrices was met (Box’s M = 30.25, *p* = 0.10). Follow-up univariate analyses indicated that age had a significant effect on perceived benefits (F(1, 151) = 7.2, *p* = 0.008, partial η² = 0.05), but not on perceived risks (*p* = 0.21). Although statistically significant, the observed effects were small. Bonferroni-adjusted group comparisons showed that relatively younger participants reported higher perceived benefits than older participants (mean difference = 0.44, SE = 0.16, *p* = 0.008, 95% CI [0.11, 0.76]). Further analysis of the relationship between age and education (χ2 = 8.93, *p* = 0.01, Cramer’s V = 0.23) showed that older participants were predominantly highly educated, holding postgraduate degrees, while younger adults mostly held undergraduate degrees.

#### The Effect of Family History of MHPs on Perceived Benefits and Risks

**Fig. 3:**
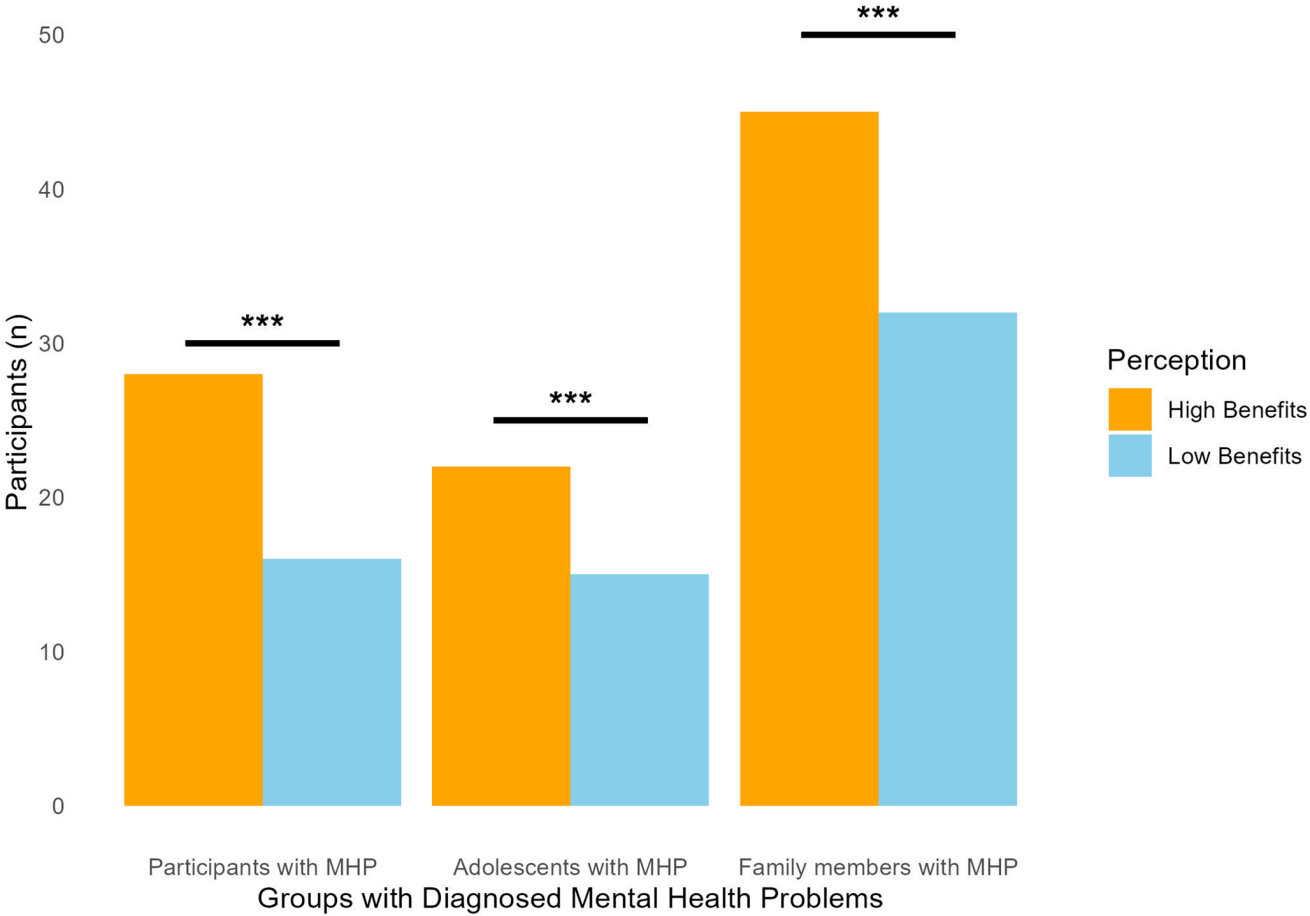
Distribution of participants with low and high benefits perceptions across three groups with diagnosed mental health problems. ***p < 0.001

**Fig. 4:**
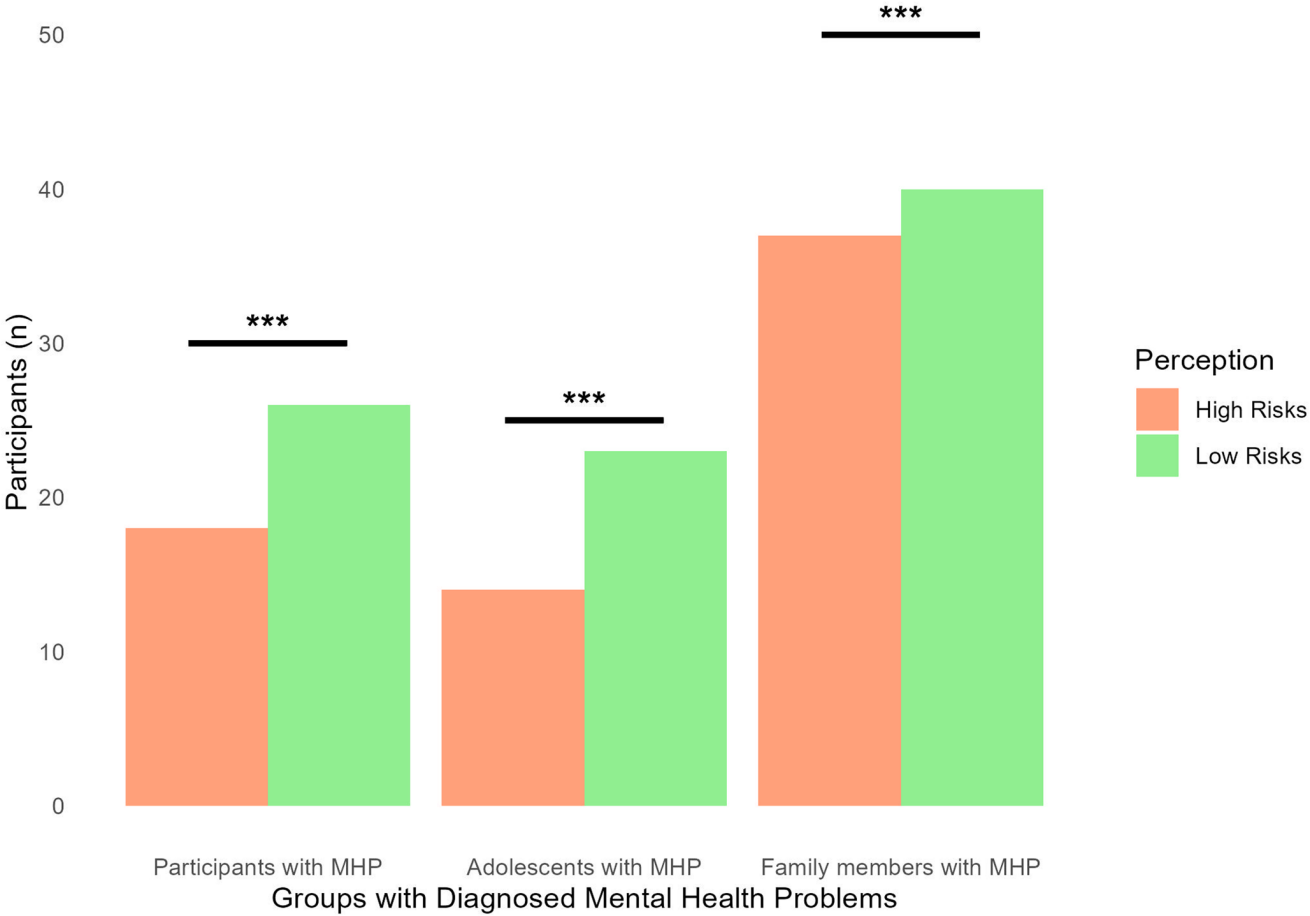
Distribution of participants with low and high risk perceptions across three groups with diagnosed mental health problems. ***p < 0.001

In the demographic section, three separate questions were included to assess whether parents, their adolescents, or other family members had been diagnosed with mental health problems. Chi-squared tests were conducted to explore differences in the perception of benefits and risks (high and low) between individuals with and without a diagnosis of MHPs. The results indicated no significant differences in perceptions between the two groups. However, independent t-tests revealed variations in opinions within the group that had received a diagnosis, as shown in Figs. 3 and 4. A significant tendency towards more perceived benefits was found among individuals with a diagnosis of mental health problems, while their perception of risks was comparatively lower.

Other demographic variables, such as gender, number of adolescents, income, and participants’ knowledge about MHPs, did not significantly affect parental perceptions of benefits or risks.

## Discussion

To our knowledge, this is one of the first quantitative studies to report parental perceptions of mental health biomarker testing among parents in Finland. Given the growing concern about the incidence of mental health problems in adolescents, biomarkers have garnered increased attention. Over the past decade, MHBs have been a topic of discussion, with numerous biomolecules showing promise as potential candidates ​(Abi-Dargham et al., 2023; Zeng et al., 2024)​. While the scientific community recognizes their potential, it is equally important to assess the perceptions of the public, as they represent the potential beneficiaries of these technologies. This study aimed to address this critical gap in the literature, as no prior studies have explored parental perceptions of MHB testing. We focused on parents of adolescents to better understand the uncertainties surrounding these perceptions. Our findings revealed that perceptions of benefits were marginally higher and concerns about risks were somewhat lower. This finding aligns with the systematic review, which emphasized that, overall, participants demonstrated a positive attitude toward genetic testing, with perceived benefits outweighing risks ​(Hann et al., 2017)​. The review underscored the primary benefit of testing as the ability to make informed decisions, while the greatest concern was the worry associated with receiving a positive result. Furthermore, in our study, parental perceptions were influenced by factors such as education, age, and a family history of mental health problems.

As most of our respondents were moderately to highly educated, the influence of individuals with lower education levels may not significantly affect our analysis due to low statistical power. As a result, the findings might be slightly skewed toward highlighting notable differences between individuals with undergraduate and postgraduate degrees. While previous studies on genetic testing have shown that higher educational attainment is often associated with more favorable attitudes (Khdair et al., 2021), our analysis indicated that participants with postgraduate education reported lower perceived benefits of MHB testing. However, because a direct measure of overall attitudes toward MHB testing was not included, it cannot be conclusively stated that our findings contradict earlier research. One possible explanation is that individuals with postgraduate education may be more inclined to anticipate potential short-term risks while placing less emphasis on long-term benefits. Additionally, in our sample, participants without a family history of MHPs were slightly more prevalent in the postgraduate group than in the undergraduate group, which may have influenced benefit perceptions. Furthermore, participants with an undergraduate degree tend to be younger than those with postgraduate education, and age-related differences may have contributed to the observed patterns. Younger participants in our study reported greater perceived benefits from MHB testing, which contrasts with findings from a previous study (Bíró et al., 2020) on public attitudes toward genetic testing. The study indicated that participants aged 40–60 found the personal benefits of testing more rewarding than their younger counterparts. Additionally, our findings indicate that having a family history of MHP led participants to lean more toward the perceived benefits of testing rather than the risks. Similar outcomes were reported in a study where first-degree relatives of Alzheimer’s disease patients highly valued the advantages of genetic testing ​(Christensen et al., 2011). Another study also highlighted greater interest in genetic testing among young adults whose grandparents were diagnosed with Alzheimer’s disease (Pavarini et al., 2021). Taken together, it appears that the history of MHPs makes parents more cautious, prompting them to explore and potentially mitigate such risks in their children by placing greater emphasis on the perceived benefits.

A key area of discussion following MHB testing is the psychological impact of the results on adolescents and their families. One of the most critical factors and limitations of genetic testing is the worry associated with obtaining positive results. However, a systematic review reported no evidence of severe psychological distress following genetic testing ​(Wakefield et al., 2016)​. The review also found no significant impact on overall quality of life. While the psychological implications of post-genetic testing have been extensively studied for various diseases ​(Oliveri et al., 2018), similar implications could arise with MHB testing. Additionally, future attitudes toward MHB testing may vary depending on the specific mental health condition and associated risk. Unlike genetic testing, which often assesses a high likelihood of disease occurrence, MHB testing for risk assessment would primarily serve as an aid for preventive tools. Its goal would be to mitigate the severity of potential disorders, ultimately reducing the economic burden of mental health conditions over time.

## Strengths and Limitations

A key strength of this study is that, to our knowledge, it is the first quantitative study in Finland to explore parental perceptions of MHB testing for adolescents. Moreover, our focus on adolescents is particularly significant, as this developmental stage is when the first incidence of mental health problems often arises, making it a critical period for early diagnosis. Our results indicated that demographic factors, like educational qualifications and age, significantly influenced perceived benefits, whereas perceived risks did not vary significantly. This finding highlights the importance of demographic factors in shaping how beneficiaries perceive benefits. This research provides a valuable example for exploring perceptions of biomarkers in other diseases, extending beyond mental health. It may also serve as a useful starting point for future longitudinal studies and reference, as MHB testing moves closer to potential clinical implementation. Additionally, our findings address the value of increasing public awareness and understanding of MHBs to advance informed decisions.

One limitation of this study is the relatively small sample size and low response rate. Consequently, the findings should be interpreted as exploratory and reflective of perspectives within this specific subgroup rather than as population-representative estimates. In addition, the original questionnaire was developed for ASD, whereas the present study adapted the items to address a broader range of MHBs. As a result, parental perceptions may vary depending on the specific conditions for which biomarker testing is proposed in the future. Currently, however, no clinically validated biomarkers exist for specific MHPs, making it challenging to assess actual benefits and risks. Accordingly, this study focused on perceived rather than actual risks. Although demographic factors influenced parental perceptions, the effect size was small. Another limitation is the geographic focus of the study, as it was conducted in Kuopio, Finland, limiting the diversity of the respondent pool. Furthermore, we studied the perspectives of parents of adolescents, without directly analyzing the perceptions of the adolescents themselves. MHB testing is still an emerging field, and no clinical tests are currently available on the market. This hinders public understanding of technology’s practical applications and its potential benefits and risks.

## Future Perspectives

While people may have some awareness of biomarkers, they possibly lack the detailed understanding required for informed decision-making. Efforts to educate the public on the benefits and risks of these technologies could enhance acceptance and utilization. Large-scale studies on public perceptions of biomarkers should be prioritized alongside initiatives to improve awareness. It is also important to explore potential risks contributing to hesitancy towards MHB testing. Additionally, public knowledge and interest in MHB testing warrant further investigation. Although minors in some countries cannot decide about their testing, their perspectives remain relevant and should be studied.

When MHB testing becomes clinically available, healthcare professionals must clearly communicate its purpose, benefits, and risks to families before testing. Tailored strategies should also be developed to minimize psychological impacts on children identified as at risk. Finally, the attitudes of psychiatrists and healthcare professionals, as key facilitators of MHB testing, should be assessed. Their views will be crucial in driving the adoption and effective implementation of these technologies in psychiatry.

## Supplementary Information

Below is the link to the electronic supplementary material.

Supplementary Material 1 (DOCX 32.8 KB)

## Data Availability

The data contains indirect identifiers for participants, which must remain confidential according to ethical approval to protect their privacy, especially given the small geographical region from which it was extracted. Access to parts of the data may be granted upon reasonable requests by researchers meeting the necessary criteria. A data-use/transfer agreement is required to ensure privacy protection and compliance with national data protection laws, with specific clauses depending on the data requested.
