# Supplementary material for: Parental Perceptions of Benefits and Risks Concerning Mental Health Biomarker Testing for Adolescents
Source: Community Ment Health J. 2026 Feb 25;62(6):1154–63. doi: 10.1007/s10597-026-01601-0 (PMC13451226; doi:10.1007/s10597-026-01601-0)
Supplement: Supplementary file 1 [file 10597_2026_1601_MOESM1_ESM.docx]

**Parental perceptions of benefits and risks concerning mental health biomarker testing for adolescents**

Kaustubh Kishor Jadhav^1^, Kirsi Kuuru^2^, Eeva Aromaa^2^, Aino-Kaisa Piironen^1^, Päivi Eriksson^2^, Katja M. Kanninen^1^, and Tommi Tolmunen^3,4*^

***Corresponding author: Tommi Tolmunen**, Institute of Clinical Medicine, Department of Medicine, Faculty of Health Sciences, University of Eastern Finland (UEF), Kuopio University.

Email:  [tommi.tolmunen@pshyvinvointialue.fi](mailto:tommi.tolmunen@pshyvinvointialue.fi)

**Submitted to**: Community Mental Health Journal

**1. Information about biomarkers and MHB testing provided to participants**

The following information was provided in the questionnaire before participants answered the questions on benefits and risks.:

*A biomarker is a substance measured from the body that gives information about a current or possible future health problem or illness.*

*Biomarker tests for mental health problems measure substances from the body that give information about a current or possible future mental health problem or mental illness.*

**2. Questionnaire design**

After deciding to utilize the questionnaire items published by Yusuf et al., it was explored whether rephrasing them could align with our research objectives. While not all questions were directly relevant to our research, 20 were retained questions to adapt to the original questionnaire (Yusuf et al., 2020). Upon reviewing its design, the questions were grouped into two categories: benefits and risks. Since MHB testing is a new and evolving technology, the questionnaire was organized into these two sections to facilitate participant understanding. The reverse-coded questions (#3, #14, #15, #16, #19, #20, #21) were categorized as assessing "risks" related to MHB testing, while the remaining questions pertaining to "benefits".

The benefit questions focused on the potential advantages the adolescent might gain from testing. In contrast, the risk questions aimed to evaluate the broader impact on the psychological well-being of the adolescent's family after testing. Three of the 23 questions (#3, #22, and #23) did not fit into either category, so they were excluded. The questions were then rephrased to categorize them as either benefits or risks of MHB testing. All reversed questions, except for #3, were included in the "risks" section, while the remaining questions, except for #22 and #23, were assigned to the "benefits" section. A 5-point Likert scale (strongly disagree to strongly agree) was used for obtaining responses and flexibility was preferred by offering a "neither agree nor disagree" option. In contrast, Yusuf et al., 2020 utilized a 4-point Likert scale. Before this section, we briefly described the meaning of biomarkers and what biomarkers are for mental health problems in layman's terms. The demographic questions included a "don't want to answer" option to offer respondents discretion in their responses.

**Table 1** Modified questions from the original questionnaire (Yusuf et al., 2020) to be adapted for our research.

| No. | Modified questions |
| --- | --- |
| B1* | Biomarker tests would help me understand why my child has a mental health problem |
| B2* | I would like my child to have biomarker tests regardless of the results |
| B3* | I would like to know the results of biomarker tests related to my child's mental health in the future |
| B4* | I would like to know the results of biomarker tests related to my own health in the future |
| B5* | Biomarker tests would give me information on how likely my other children are to develop a mental health problem |
| B6* | Biomarker test results would help me decide if I want to have more children |
| B7* | I would like to have a biomarker test during pregnancy to find out how likely the child is to develop a mental health problem |
| B8* | Biomarker test results would help me prepare for my child's future |
| B9* | Biomarker tests would help me stay informed about future treatment options |
| B10* | Biomarker test results would help me take better care of my child and family |
| B11* | Biomarker test results could help prevent my child from developing other health problems |
| B12 | The results of biomarker tests and their potential to prevent mental health problems would put my mind at ease about my other children's risk of illness |
| B13 | Biomarker test results would make me more watchful for my other children's symptoms |
| B14* | Biomarker tests would give me information about other mental health problems my child may develop |
| R1* | Biomarker test results can undermine my freedom to decide about my child's health |
| R2* | Biomarker test results could stigmatize my family |
| R3* | Biomarker test results can cause problems between me and my (ex) partner |
| R4* | After biomarker tests, I may feel guilty that I may have passed on a biological risk to my child |
| R5* | Biomarker test results may leave me living in uncertainty |
| R6* | Biomarker test results may make me focus on mental health problems instead of what life has to offer |

*mandatory questions

The word ‘child’ is used in our questionnaire for better translations and understanding in Finnish, instead of using adolescents. Questions B1-14 deal with the benefits of MHB testing, whereas questions R1-6 deal with the risks of MHB testing. The English version of the questionnaire was translated into Finnish using Lingsoft Ltd. translation services. Native Finnish-speaking co-authors further refined the translation.

**3. Distribution**

Participants received postal documents containing survey details, participant rights, informed consent, and the privacy policy statement. The documents included a link and QR code directing them to the online questionnaire.

**4. Missing values**

All demographic items were mandatory; however, respondents were given the option to select ‘don’t want to answer’ for gender, income, and family history of mental health problems. Responses selecting this option were subsequently coded as missing for analyses. Missingness for these variables was low (<5%).

**5. Accessing the perceived knowledge**

In the demographics section, a question was designed to assess participants' perceived knowledge of MHPs rather than their actual knowledge.

"What is your knowledge about mental health problems?"

Participants were provided with four response options to this question: no knowledge, some knowledge, substantial knowledge, and extensive knowledge of MHPs.

**6. Removal of questions after EFA**

Questions B1 and B5 were excluded from the analysis due to their factor loadings being below 0.5. Questions B6 and B7 loaded onto a different factor (other than Factor 1), with loadings exceeding 0.7, and were also found to lack content validity. Similarly, questions B2, B3, and B4 loaded onto a different factor; however, only B4 was excluded, as it lacked content validity in relation to MHB testing for adolescents. The remaining questions (B2 and B3) were retained in the questionnaire due to their appropriate content relevance.

**7. Piloting**

The readability and unambiguity of the questionnaire in Finnish were further enhanced by conducting a pilot survey with a group of parents (n = 14) who were not involved in the actual survey. In addition to the main questionnaire, a feedback section was included to gather information on the time required to complete the questionnaire, the perceived difficulty of understanding the questions, and any specific questions participants found particularly challenging to answer. Feedback from the pilot survey was used to improve and optimize the readability and clarity of the questionnaire.
